# Supplementary material for: Are carbon emissions trading and green financial instruments synergistic? -Comprehensive quantitative research based on content analysis
Source: PLoS One. 2024 Mar 7;19(3):e0298601. doi: 10.1371/journal.pone.0298601 (PMC10919601; doi:10.1371/journal.pone.0298601)
Supplement: S1 Data — (ZIP) [file pone.0298601.s001.zip › Data/地级市Z_F工作报告.zip!/╜¡╦╒/╒≥╜¡╩╨/└┤╫╘╣1⁄2╓┌║┼í╛┬φ┐╦╩2╛▌═°í┐_2019.docx]

2018年工作回顾

预计实现地区生产总值4050亿元，增长3%；一般公共预算收入301.5亿元，增长6%；社会消费品零售总额1360亿元，增长6%；城乡居民人均可支配收入达49020元、24660元，分别增长8%、8.5%；城镇登记失业率1.8%；完成省下达的节能减排约束性指标。

一、面对转型升级的压力，坚持产业强市不动摇，经济运行质态得到提升

重大项目取得突破。175个市重点产业项目完成年度投资计划，其中制造业投资占比达68.6%。新签约亿元以上项目167个，其中制造业项目占比达76.6%，北汽麦格纳高端智能纯电动汽车、长城汽车零部件生产基地、孚能科技制造基地、协鑫电动生态科技产业园、蝙蝠飞机制造厂等一批投资量大、带动力强的先进制造业项目落地。

恒大童世界、康缘中华养生谷入选省级重点旅游项目，好未来镇江教育基地项目签约落户。5家企业入选中国民营企业500强，4家企业成为省质量标杆企业，新增8家国家“两化融合”贯标试点企业，创成6家省级示范智能车间。“戴庄经验”在全省推广，新增高标准农田19.7万亩、省级农业龙头企业6家，绿色优质农产品占比达35%。

园区承载能力提升。镇江经济技术开发区创成省级生态工业园，简政放权创业创新环境评价位居全省前列，国家大学科技园创成省级生产性服务业集聚示范区，国家级开发区综合考评位列全国第22位，较上年提升20位。赋予镇江高新区直接审批、执法事项265项，中集智能物流装备产业园、美的智能装配产业园、金牛山片区合作开发等项目落户，入选国家“双创升级特色载体”，并创成省知识产权示范区。丹阳经济技术开发区综合考评位居省级开发区榜首，创成省级出口农产品示范区。句容绿色新能源小镇考核居全省首批特色小镇第3位，创成省级现代农业产业示范园。扬中省级高新区正式获批，创成国家智慧电气高新技术产业化基地。丹徒经济开发区通过国家循环经济标准化试点园区验收。扬中智慧电气小镇、镇江新区再生医学小镇入选第二批省级特色小镇。

改革开放不断深化。扎实推进供给侧结构性改革，完成去产能项目60个，化解水泥产能180万吨；开展化工企业“四个一批”专项行动，关停化工企业38家，化工企业入园进区率达54%。推进农村各项改革，农村土地承包经营权确权登记颁证工作基本完成，83%的村完成农村集体产权制度改革，287个村完成会计服务中心委托代理。润州成为全省新一轮服务业改革试点区域。深化“放管服”改革，构建市县镇村四级政务服务体系，“3550”目标基本实现，企业开办用时压缩45%，市级全程不见面审批率达99.6%。进出口贸易创历史新高，对“一带一路”沿线市场出口达25.9亿美元，增长23%；与法国贝尔热拉克城市聚合体、澳大利亚洛克汉普顿市建立友城关系，举办镇江市与韩国益山市缔结友城关系20周年、“第四届东盟文化之旅”等系列活动。

创新动能明显增强。市技术交易市场网络平台投入运营，新增国家级高新技术企业122家，高新技术产业产值占比达49.5%，位居福布斯中国最具创新力城市第18位；2家企业入选“独角兽”企业榜，新增省级“科技小巨人”企业6家、上市挂牌企业117家，1家企业获得中国专利金奖，京口获评“国家知识产权强县工程示范区”。新增省级企业技术中心9家、省级企业工程技术研究中心10家、省级众创社区5家和孵化器5家。培育引进“国家杰出青年”等人才21人、省“双创计划”等人才98人、市“金山英才”计划118人。成功承办第四届江苏技能状元大赛，7人荣获“技能状元”称号，取得历史性突破。侯卫东官场笔记

营商环境持续优化。定期召开企业家座谈、政银企对接等交流活动，创成企业网络服务国家级示范平台。完善外商“一卡通”优惠服务。打击商标侵权违法行为，深化全省“网络市场监管与服务示范区”创建。全面落实各项减税降费政策，全年减免各项税费106亿元；推进口岸提效降费，率先公示收费清单，整体通关时间压缩1/3。在全省率先实现综合金融服务平台上线运行，帮助1187家企业获得授信105.3亿元、贷款76.5亿元。

二、面对环境治理的挑战，切实加强整改不懈怠，生态环境质量得到改善

开展专项行动。扎实开展“263”“116”专项行动，系统推进水气土治理，中央环保督察“回头看”和省环保督察交办信访件全部办结。打好蓝天保卫战，实施大气污染防治项目375项，全市PM2.5平均浓度51微克/立方米，空气优良天数达标率70.1%。打好碧水保卫战，全面落实河（湖）长制，开展河湖“三乱”、长江岸线保护等专项整治行动，整治入河排口215个，关闭违规畜禽规模养殖场149家，长江镇江水域非法采砂全面禁绝，完成江心洲水源地达标建设，长江豚类自然保护区问题整改到位，完成四明河、一夜河、虹桥港、御桥港、西团结河5条黑臭水体治理任务，预计国考、省考断面水质优III比例分别为87.5%、80%，重点江河湖泊水功能区水质达标率90%以上。打好净土保卫战，积极开展土壤污染治理与修复试点，出台《镇江市污染地块环境管理联动方案（试行）》，完成全国污染源普查工作。

强化片区整治。西南片区突出粉尘治理，2家重点企业完成环保设施升级改造，非法采矿全面禁绝，废弃宕口、关闭企业复垦复绿有序推进。东部片区突出综合整治，完成15家工业企业污染治理，关停“散乱污”企业148家，解决长江沿岸雨污管网混接、固废倾倒等问题28个；稳妥推进镇江新区化工园区、索普化工基地周边居民搬迁工作。高资片区突出环境提升，关停焦化生产线，关停各类“散乱污”企业58家。

厚植生态本底。启动长江经济带战略环评及“三线一单”划定编制工作，完成生态红线优化调整。加大土地复垦力度，新增耕地1.7万亩，完成成片造林1.5万亩，抚育森林6万亩，林木覆盖率达25%，自然湿地保护率达54%。全市12个镇、8个村创成首批省生态文明建设示范镇（村），丹徒区通过省级生态文明建设示范区考核验收。应邀参加第24届联合国气候变化大会，成功举办“2018国际低碳（镇江）大会暨江苏省生态环境高质量主题论坛”。提升生态安全防护能力，危险废物设施投运率90%以上。

三、面对矛盾风险的叠加，坚决守住底线不畏难，社会稳定大局得到巩固

坚决化解重大风险。综合采取严控政府投资、盘活闲置资产、加强省市国企合作、压缩一般性支出等措施，地方政府隐性债务得到有效控制。推进“破圈解链”工作，签署政银企联合公约，协调化解骨干企业融资风险，重点地区担保圈风险总体可控；开展防范金融风险专项行动，严厉打击非法集资和恶意逃废债，重点法人银行风险处置取得明显成效，金融生态持续优化。

坚决解决积存问题。盘活“四集团一中心”闲置房产230万平方米，盘活率达88%。实施市区“集中拆违行动”，拆除违法建设45.2万平方米。推进集约节约用地，建成“慧眼守土”动态智能监管系统，整改存量违法用地1.8万亩，处置各类闲置土地4290亩。研究处置市中小企业信用担保中心、“退城进区”政策废止等历史遗留问题，依法依规完成韦岗铁矿、胜华船厂、联合水泥等资产处置，妥善处置7家国有煤矿企业安全环保、综合利用等问题，化解37个小区3.4万户产权证办理难题，消除逾期安置4540套，完成国有企业职工家属区“三供一业”分离移交协议签订。

坚决维护和谐稳定。开展社会矛盾排查和风险防控，圆满完成重要节点时期信访维稳任务，成功处置专项维稳事件。开展重点行业安全生产专项整治行动，事故起数和死亡人数保持“双下降”。成立全国首个“安全守护联盟”，形成多方参与的社会化防灾减灾救灾格局。开展“扫黑除恶”“三打击一整治”等专项行动，深化完善基层网格化社会治理机制得到中央领导肯定，公众安全感保持全省前列。

四、面对美好生活的期待，坚持精准施策不停步，惠民发展实效得到增强

在城乡建设上，强化规划龙头作用，市区1088平方公里规划全覆盖，“多规合一”取得实质性进展，中心城区380平方公里、33个单元控制性详细规划编制完成。安排财政资金3亿元，运用“共同缔造”理念，推进中营、大市口北等五个老旧小区改造，完成危房和空斗墙房屋改造2.37万平方米，解危搬迁八摆渡、江小圩受淹片区群众；建立征收拆迁“市统筹、区实施”工作机制，市区完成棚户区改造170万平方米。海绵城市建设完成试点任务。高校园区建设顺利推进，江苏大学京江学院新校区交付使用。镇丹高速、句容西部干线建成通车，南沿江高铁、宁句城际轨道交通、镇江高铁南站优化提升工程正式开工，连淮扬镇铁路镇江段、五峰山过江通道及其接线高速公路加快推进，江滨路交叉口、西府街等断头路、瓶颈路完成改造；长江南京以下12.5米深水航道工程镇江段全面完成，扬中长江崩岸段干堤复建和岸线应急治理、九曲河整治工程基本完成；智慧城市综合运行监管平台基本建成，工业云平台、智慧信用上线运行。市区建设市政燃气管道79公里，改造老旧燃气管道83公里，完成上党液化天然气储配站二期扩建工程；新建改造供水管网40.4公里、污水管网30.3公里；建成世界最大规模电网侧储能电站群。农村环境持续改善，提档升级农村公路200公里，改造农桥20座，新增80个美丽宜居村，3个省级特色田园乡村基本建成。

在城市管理上，深化更高水平文明城市创建，扎实推进城市治理与服务十项行动，开展露天烧烤、电动车违法行为等专项整治，加强交通堵点治理，对拥堵路段过境货车有序分流疏导。全市257个居民小区、424个党政机关开展垃圾分类试点，主次干道垃圾分类投放设施覆盖率达80%。新增优化公交线路61条，市区实现公交扫码乘车移动支付全覆盖，完成镇村公交智能化改造。推行机关、企事业单位停车位对外开放，新增停车泊位1万个，道路临停泊位“互联网+地磁”系统上线运行。

在基本保障上，深化产业扶贫、电商扶贫、健康扶贫等行动，全市54个经济薄弱村达标，3300余户建档立卡低收入人口实现脱贫，达标率超过80%。实施“全民参保计划”，城乡困难人群纳入保障体系，社保覆盖率98%以上，低保标准提高到每人每月710元，退休人员养老金实现“十四连增”。优化职工医保统账结构，改善群众医保待遇；完善“医疗保险+医疗救助”与慈善助医衔接机制，实施医疗救助31.6万人次；开通“电子医保卡”和医保移动支付平台。在全省率先构建社会救助云平台。新建老年人日间照料中心5个、助餐点100个、居家养老服务中心15个。

在公共服务上，完善就业创业服务体系，新增城镇就业8.4万人，扶持创业1.33万人，创业带动就业6.04万人。出台市区义务教育布局规划，实施“八校联动、三大工程”，南师附小镇江小学、镇江心湖高级中学投入运营，苏州外国语学校官塘校区项目签约落户，学前教育体制改革获“全国教育改革创新优秀奖”。建立642支家庭医生签约服务团队，开设103个全-专联合门诊，建成20家康复联合病房，重大疾病分级诊疗体系不断健全。5个菜市场建成开业。创成市级“放心肉菜示范超市”8家，全市学校、养老机构“明厨亮灶”覆盖率分别达90.1%和98.5%。建成省级村（社区）基层综合文化服务中心277个、农村文化礼堂236个，镇江民间文化艺术馆新馆对外开放，江苏电力历史博物馆建成开馆，延陵村农家书屋获评“全国示范农家书屋”。省级双拥模范城市再创“满堂红”。成功举办2018砂板乒乓球世界杯、江苏省第十届残运会等重大赛事，镇江籍运动员在亚运会、全国击剑锦标赛上勇创佳绩。政府法制、侨务、行政学院、人口计生、民族宗教、机关事务等工作取得新成绩，档案、地方志、防震、气象、供销、科普、老龄、妇女儿童、关心下一代、见义勇为、残疾人、红十字等事业取得新进展，国家安全、国防教育、人民武装、人民防空、援藏援疆援陕等工作取得新成效。

在总结成绩的同时，我们清醒地认识到，发展中还存在一些深层次矛盾和问题：一是部分指标欠序时，受宏观经济下行、消化过高基数等因素影响，地区生产总值、社会消费品零售总额、固定资产投资三项指标未能完成人代会确定的目标。二是产业结构偏重，先进制造业发展还不充分，生态环境仍存在许多短板和问题。三是影响社会稳定的隐患依然较多，地方政府隐性债务、非法民间金融活动、安全生产等领域的风险隐患不容忽视。四是民生领域还有不少短板，城乡发展还不平衡，基础设施建设仍然滞后，公共服务水平有待进一步优化。五是政府职能转变还不到位，服务效能还不够高，形式主义、官僚主义一定程度上仍然存在，营商环境需进一步改善。对此，我们一定高度重视，采取有力措施，切实加以解决。

2019年工作总体要求

2019年是新中国成立70周年，也是高水平全面建成小康社会的关键之年。今年政府工作的总体要求是：以习近平新时代中国特色社会主义思想为指导，全面贯彻党的十九大和中央经济工作会议、省委省政府及市委的决策部署，坚持稳中求进工作总基调，坚持新发展理念，坚持推动高质量发展，坚持以供给侧结构性改革为主线，坚持深化市场化改革、扩大高水平开放，继续打好三大攻坚战，统筹做好稳增长、促改革、调结构、惠民生、防风险工作，保持经济持续健康发展和社会大局稳定，在高质量发展新征程上奋勇前进，打好高水平全面建成小康社会决定性基础，以优异成绩庆祝中华人民共和国成立70周年。

今年经济社会发展的主要预期目标是：地区生产总值增长6.5%以上；固定资产投资增长6.5%左右；一般公共预算收入增长4.5%左右；社会消费品零售总额增长7.5%左右；研发经费支出占地区生产总值比重2.55%；城乡居民人均可支配收入与经济增长同步；全面完成生态环保约束性指标。

做好今年工作，完成全年任务，必须按照市委七届八次全会的部署，进一步把思想、力量、智慧汇聚到高质量发展上来：深刻理解高质量发展的内涵，坚决落实高质量发展的要求，积极抢抓高质量发展的机遇，始终坚定高质量发展的信心。

2019年重点工作

做好今年工作，围绕高质量发展和“六个稳”要求，重点在七个方面狠下功夫、狠抓关键。

一、坚定不移推进产业强市

产业是高质量发展的基石，集中力量推动产业结构转型升级，加快补齐制造业短板，着力构建现代产业体系。

扩大有效投入。狠抓项目建设，加快推进北汽麦格纳高端智能纯电动汽车、孚能科技制造基地、长城汽车零部件生产基地等178个市级重点产业项目，当年完成投资400亿元。强化项目招引，新签约亿元以上产业项目160个。加大技术改造，鼓励引导企业积极运用新装备、新工艺、新技术、新材料，开展高端化、智能化、绿色化改造；依托工业云平台，推进企业“设备联网”工程，新增省级星级上云企业30家。推动第五代移动通信网络试点部署，加强人工智能、工业互联网、物联网等新型基础设施建设，积极发展共享经济、数字经济、生物经济、现代供应链等新产业新业态新模式。

做强特色产业。实施高端装备、新材料升级行动计划，推进“智能制造三百工程”，新增省级示范智能车间（工厂）3家、省首台（套）重大装备5个；壮大高性能纤维及复合材料、新能源等战略性材料产业规模，加快布局增材制造、石墨烯等新材料产业，落实新材料首批次应用保险政策；提升航空航天国家级新型工业化示范基地功能，争取重大军民融合项目落户。深化国家全域旅游示范区创建，推进三山南山、茅山等景区市场化改革，旅游总收入突破千亿元。探索“工业+物流+金融”融合模式，新增省级重点物流企业（基地）1家以上。积极招引外地金融机构落户，做大做强本地法人金融机构，新增上市挂牌企业20家。培育新消费热点。

狠抓政策落地。保持政策的连续性和稳定性，严格执行国家和省减税降费政策；全面落实省“降低企业负担28条”，出台我市降低企业负担政策措施。完善政策执行方式，执行政策不搞“一刀切”。树立“亩产论英雄、效益论英雄、能耗论英雄、环境论英雄”的政策导向，对企业评价分类、精准引导，稳步推进企业优胜劣汰。

壮大民营经济。坚决贯彻“两个毫不动摇”，切实支持民营企业发展壮大，提振信心、保持定力，让民营企业放胆投资。全面破除民营企业发展障碍，降低企业成本，减轻企业负担，让民营企业放开搞活。大力保护民营企业合法权益。积极构建新型政商关系，在靠前服务上体现“亲近”，在规范服务上做到“清白”，让民营企业放手发展。

二、坚定不移深化改革开放

以改革造就新气象，以开放带来新活力，以创新打开新空间，推动发展质量更高、竞争力更强。

加大改革强度。深化“放管服”改革，推进“不见面审批”改革。推动国资国企改革，完善国有资产管理体制，实现从管资产向管资本转变；积极引进战略投资者，实施兼并收购、资产重组，提升国有上市公司资本运作和抗风险能力。加快产业集团改革，整合市属产业集团相近产业和资源，积极稳妥有序推动产业集团转型。完成政府机构改革，推动下蜀镇、新坝镇强镇扩权。实施国民经济统一核算改革，完成第四次全国经济普查。

扩大开放程度。贯彻落实国家扩大开放新举措和全省对外开放大会精神，抢抓放宽市场准入机遇，提升高端装备制造、新材料等重点产业外资贡献度，推动外资企业增资扩股，制造业到位外资6.5亿美元。推进通关便利化，落实口岸提效降费要求，培育外贸新业态，推动跨境电商和外贸综合服务企业融合发展，谋划建设外贸综合服务中心。壮大进口经营企业，放大中国国际进口博览会效应，扩大先进设备和技术进口。发挥中信保“镇江统保平台”作用，帮助企业降低贸易风险，支持企业开拓“金砖国家”和“一带一路”市场。

提升创新力度。充分发挥开发园区创新主阵地作用，加快推进苏南自主创新示范区建设，支持丹阳创建国家级经济开发区、扬中创建国家级高新区、句容和丹徒创建省级高新区。加强园区市场化运作，支持镇江高新区建设国家创新型特色园区并和丹徒合作共建产业园区。落实省“科技改革30条”和市“科技改革20条”，加强规模以上工业企业研发机构建设，实施高新技术企业培育计划，认定国家级高新技术企业200家。加大对中小企业创新支持力度，培育壮大技术交易市场，支持中小微企业向驻镇高校、科研院所等购买技术服务，加强知识产权保护和运用。优化升级“金山英才”计划，实施柔性引才，壮大人才经纪人队伍，新增高层次产业领军人才200名、高技能人才6000名以上。

三、坚定不移提升品质内涵

牢固树立“精明增长”“紧凑城市”理念，深入推进“多规合一”，注重内涵提升，建设幸福感强的宜居宜业城市。

构建综合交通体系。完成连淮扬镇铁路镇江段桥梁主体工程建设，开展五峰山过江通道及其接线高速公路路基和桥梁施工，加快实施宁句城际轨道交通、南沿江高铁等项目，积极推进润扬第二过江通道、宁马宁扬城际、连镇铁路南延接入南沿江高铁和宁杭高铁、宁句城际延伸至茅山等前期可行性研究，加快建设镇江东站交通枢纽。推动大港港区与外部高速公路、铁路的顺畅衔接，建设铁路集装箱集散地，打通临港交通“最后一公里”。完成团山路西段城市化改造，开工建设蚕桑路，启动谷阳路桥拓宽及安全隐患整治工程。

补齐公共设施短板。持续推进“城市双修”，着力改善老旧城区人居环境，对大市口等老城区实施成片品质提升，统筹推进棚户区、老旧小区改造以及危旧房解危，修缮直管公房8000平方米，完成1万平方米街巷改造、10个积水区整治。推动零星地块、闲置地块品质提升和功能塑造，为市民提供更多公共空间。加快市区缺陷污水管网和谏壁、丹徒老集镇等空白区域规划改造，逐步实现污水管网全覆盖、全收集、全处理。提升水电气保供能力，完成大港水厂一期工程，新增日供水能力20万立方米，实施高校园区供水设施、城乡供水管网改造等项目；加快推进500千伏大港变电站建设；推广使用管道天然气，新建镇江大港至姚桥高压燃气管道14公里，改造老旧燃气管道80公里，建设高资、姚桥高中压站和世业洲、高桥液化天然气综合站。

加强城市精细管理。深化露天烧烤、占道经营、违章停车等专项整治，加大共享单车管理、环卫保洁、防违拆违等工作力度，推动文明城市长效管理。新增135个垃圾分类试点居民小区，加快餐厨废弃物处置二期、丹阳市生活垃圾焚烧发电厂建设，构建生活垃圾分类投放、收集、收运和处理体系。完善智慧镇江“1+1+N”体系，建设全市统一的人口、法人、空间地理等基础数据库，城市综合运行监管平台、智慧城管、智慧旅游、大数据共享交换平台上线运行，进一步提升城市治理科学化、智慧化、精细化水平。

四、坚定不移推动绿色发展

深入落实生态环境保护责任，着力控制污染源头，狠抓末端排放治理，促进生态环境质量持续改善。

整改突出问题。深入实施“263”“116”专项行动。持续开展煤电企业、“散乱污”企业、柴油车尾气、工地扬尘等领域集中整治。完善河（湖）长制，全面完成集中式饮用水源地安全隐患整治任务，建成区黑臭水体基本消除。推进土壤污染治理，加快固体废物处置设施建设。

加快绿色转型。推动产业绿色发展，深化传统产业“八个一批”工程，培育市级以上绿色工厂10家、绿色园区1家，化工企业入园进区率提高到60%。促进能源结构优化。推进绿色港口和绿色公路建设。深化农业面源污染治理。推进低碳城市建设，推动国际低碳（镇江）大会市场化运作。

增加生态供给。完成“三线一单”划定工作，建立分区管控体系。加大长江岸线生态保护力度。加强生态修复，加快岸线整治、宕口治理，完成成片造林面积8000亩，推进主城区山体“一山一景”改造、古运河水环境综合整治，打造“亲山”“滨水”独特城市风貌。

健全治理机制。深入推进排污权有偿使用和交易。指导帮助企业制定环境治理解决方案。依法打击生态环境违法犯罪行为。

五、坚定不移实施乡村振兴

坚持农业农村优先发展，大力弘扬“亚夫精神”，全面推广“戴庄经验”，加快推进农业农村现代化。

壮大乡村产业。优化“三带三区”空间布局，壮大优质粮油、特色园艺等优势产业，新增高效设施农业面积1.5万亩、渔业面积1000亩。推进丁庄葡萄、扬中河豚等特色小镇建设，新增省级以上休闲农业示范点4个，农业电商网上销售额增长20%以上。扩大绿色农产品基地规模，规划建设高效生态农业示范基地30个，新培育国家级和省级示范社30家，绿色优质农产品占比达到40%；全面完成粮食生产功能区和重要农产品生产保护区划定工作，新建高标准农田8万亩。

优化乡村环境。统筹生产设施、生活服务设施、社会事业设施等规划，鼓励农民和农村集体经济组织自主开展村内基础设施建设。深化特色田园乡村建设，完成174个规划发展村庄和居民点生活污水治理，新增100个美丽宜居村庄、20个绿化示范村。推进“四好农村路”建设，提档升级农村公路100公里，改造农桥10座。建设农村生态河道20条，新增“水美乡村”15个。

深化乡村改革。深化农村集体产权制度改革，力争全市90%以上的村完成改革任务，规范农村产权交易市场，推动农村集体资产保值增值。深化农村土地制度改革，推进农村宅基地“三权分置”试点，加快推进房地一体的农村集体用地和宅基地使用权确权登记工作，支持以出租、联营、入股等形式，盘活利用闲置农房及宅基地。深化农业保险经营模式改革。统筹推进供销合作社、农村水利、集体林权制度改革，增强为农服务能力。

培育乡村人才。壮大家庭农场、农民专业合作社等新型经营主体，培育新型职业农民8000人。完善基层农技、农机推广体系，全市“一村一所”占比达80%。引导鼓励大学生、农民工返乡就业创业，扶持一批农业职业经理人、经纪人，培育一批乡村工匠、文化能人、非物质文化遗产传承人。

全面精准脱贫。开展省定标准脱贫人口“回头看”，严格落实“两不愁三保障”要求，完成脱贫攻坚各项目标任务。支持经济薄弱村发展特色产业，增强发展内生动力；完善新型经营主体与低收入农户利益联结机制，带动贫困人口增收致富。加强精准管理和阳光监管，制定“一村一策”“一户一策”任务清单。强化健康扶贫。

六、坚定不移维护社会稳定

始终把防范化解重大风险作为重中之重，坚持底线思维，采取过硬措施，营造和谐、安全、稳定的发展环境。

坚守金融安全底线。按照“坚定、可控、有序、适度”要求，严控政府投资项目，压减政府一般性支出，坚决守住不出险底线。深入实施“清风行动”和金融生态环境“八城同创”。

有效化解社会矛盾。健全社会矛盾排查预警、重大决策社会稳定风险评估和矛盾纠纷多元化解机制，有效预防和化解矛盾纠纷。积极推进社会治理重心下移，提高社会治理水平。推进公安大数据指挥服务中心和执法办案基地、实战训练基地建设，继续开展“扫黑除恶”专项斗争。

维护社会公共安全。加强食品药品安全监管，坚决防范重特大食品药品安全事故。严格落实安全生产责任制，全市生产安全事故起数、死亡人数保持“双下降”。持续开展电动车违规行驶、渣土车超载超速等专项整治，防范处置公共交通领域突发极端事件。健全应急管理机制，加快预警发布系统和民间救援体系建设，不断提升公众安全感。

七、坚定不移持续改善民生

聚焦群众关注的热点难点问题，精准发力、持续攻坚，不断增强广大群众的获得感和幸福感。

稳定就业创业。把稳就业摆在突出位置，新增城镇就业7万人，扶持大众创业7000人，创业带动就业3.5万人，争创2家省级示范基地。建立健全终身职业技能培训制度，全年培训城乡劳动者就业技能1.5万人。继续实施“万名高校毕业生留镇行动”，全年培养引进本科以上人才1.6万人。

强化社会保障。深入推进社保制度改革，落实机关事业单位养老保险制度改革政策，城乡社保覆盖率保持98%以上。继续做好城乡居民和灵活就业人员社保费征缴工作。深化医保支付方式改革，稳步推进医保“按疾病诊断相关分组”收付费试点；打击欺诈医保基金行为，防范基金风险。大力发展社会慈善事业，完善多层次救助体系，全面落实残疾人“两项补贴”及成年无业重度残疾人“单人保”政策，建成市残疾人创业就业孵化基地。

坚持教育优先。完成市区学前教育布局规划修编工作，优化普惠性学前教育资源配置。探索集团化办学模式，深化教师“县管校聘”改革，扩大优质教育资源受益范围。实施高中办学体制改革，推动高中教育优质多样发展，促进普教、职教渗透融合。启动第四中学迁建工程。探索数字化督导、第三方督导，促进教育“管办评”分离。加强校外培训机构专项整治，完善长效监管机制。加快推进高校园区建设，江苏科技大学、市委党校等6所学校全面入驻。

建设健康镇江。完善市区医疗设施布局规划，优化医疗资源配置。深化公立医院综合改革，完善大医院与基层医疗卫生机构共建共享机制。引进和培育医生集团，增加医疗服务供给。加强家庭医生工作室建设。推进健康镇村建设，争创2个省级健康镇、5个省级健康村。开展应急救护“百万培训”。

促进文化繁荣。完成市区公共文化布局规划，国家公共文化服务体系示范区创建通过中期评估，提升镇（街道）文化广场15个、村（社区）文化广场37个。开展金山艺术展演月、民俗文化周等文化惠民活动，继续办好长江国际音乐节。加大历史文化名城保护力度，推进大运河文化带建设，实施京口闸遗址、焦山摩崖石刻、殷氏六房旧宅保护工程，加强传统表演艺术类、手工技艺类、民俗活动类等非物质文化遗产保护传承。

推动全民健身。组织群众喜闻乐见的足球、篮球、乒乓球等“十大业余联赛”，开展全民健身活动200场（次）以上。打造世业洲体育健康公园。进一步提高公共体育场馆使用效率，提供高质量体育服务。

今年，将努力办好事关民生福祉的10件实事。

1.优化医疗保障。试点“中医经典病房”建设，在村卫生室、社区卫生服务站建设中医阁100个；推进医保政策市级统筹，优化职工基本医疗保险门诊和住院统筹制度，整合补充医疗保险制度，提高医保“特药”报销比例10个百分点。

2.优化养老服务。新建5个老年人日间照料中心，加快建设标准化及以上社区居家养老服务中心，推进农村敬老院“公建民营”；深化医养融合，在社区日间照料中心嵌入医疗服务。

3.优化教育布局。完成市区三中改扩建、江南学校扩建、实验初中迁建工程和中山路小学、李家大山小学改扩建工程，建设丹徒茅以升学校，异地新建句容市高级中学，创建40所智慧校园。

4.优化体育设施。建成健康路市民体育公园并对外开放，建成镇（街道）多功能运动场10个、笼式足球场5个、健身路径80条。

5.优化菜场建设。筹划建设农产品批发市场，新建改造4个菜市场，加强菜市场长效管理综合考评。

6.优化公厕环境。提高标准、优化布局、完善功能、创新管理，新建环卫公厕18座，改造环卫公厕56座。

7.优化公交出行。探索主要交叉口公交车信号优先，市区新购置190辆新能源公交车，新辟、优化公交路线20条。

8.优化停车管理。新增1万个停车泊位，推进“互联网+停车”全覆盖，实现扫码收费、精细管理，提高停车服务智能化水平。

9.优化道路通行。打通宗泽路、汝山路等断头路、瓶颈路，改善演军巷、仙鹤巷等道路微循环；综合采取科学调度、合理分流等措施，缓解南徐大道等城市拥堵路段。

10.优化文化服务。完成11个茅山革命老区经济薄弱村综合文化服务中心强基层任务，开展全民艺术普及惠民文化活动500场。

加强政府自身建设

新的一年，我们将牢固树立“四个意识”，坚定“四个自信”，坚决践行“两个维护”，主动作为、开拓进取，努力建设人民满意的服务型政府。敢担当，提效能，重法治，讲诚信，促清廉。
